# Mucosal-Associated Invariant T cell in liver diseases

**DOI:** 10.7150/ijbs.39016

**Published:** 2020-01-01

**Authors:** Yujue Zhang, Derun Kong, Hua Wang

**Affiliations:** 1 Department of Gastroenterology, The First Affiliated Hospital of Anhui Medical University, Hefei, 230032, China;; 2 Department of Gastroenterology, Fuyang Hospital of Anhui Medical University, Fuyang, Anhui 236000, P.R. China;; 3 Department of Oncology, The First Affiliated Hospital of Anhui Medical University, Hefei, Anhui, 230032, China.

**Keywords:** alcoholic liver disease, autoimmune liver disease, liver cancer, MAIT cells, non-alcoholic liver disease

## Abstract

Mucosal-associated invariant T cells (MAIT cells) are a new population of innate immune cells, which are abundant in the liver and play complex roles in various liver diseases. In this review, we summarize MAIT cells in the liver diseases in recent studies, figure out the role of MAIT cells in various liver disease, including Alcoholic liver disease, Non-alcoholic liver disease, Autoimmune liver diseases, Viral hepatitis and Liver Cancer. Briefly, MAIT cells are involved in anti-bacteria responses in the alcoholic liver diseases. Besides, the activated MAIT cells promote the liver inflammation by secreting inflammatory cytokines and produce regulatory cytokines, which induces anti-inflammatory macrophage polarization. MAIT cells participate in the liver fibrosis via enhancing hepatic stellate cell activation. In viral hepatitis, MAIT cells exhibit a flawed and exhausted phenotype, which results in little effect on controlling the virus and bacteria. In liver cancer, MAIT cells indicate the disease progression and the outcome of therapy. In summary, MAIT cells are attractive biomarkers and therapeutic targets for liver disease.

## 1. Introduction

Liver is an important immune organ and maintains the steady state of the homeostasis. Besides, it receives 75% of blood supply from the gastrointestinal tract through the portal vein, which plays a unique role in the pathogen resistance system in the blood circulation 1. When the blood flow enters the liver, it passes through the network of innate and adaptive immune cells in hepatic sinusoid. Thus, the liver can be considered as a firewall to prevent the infection invasion into the systemic circulation. When infected with various pathogens, the liver innate cells secrete variety of cytokines, forming the first line of defense. With the progress of the diseases, the acquired immune cells play a dominant role in anti-infectious diseases. In humans, MAIT cells are widely distributed in the body, especially in liver which constitute up to 10-50% of T cells^2^. MAIT cells can be activated by riboflavin metabolites derived from microorganisms through non- polymorphic MHC class I- related (MR1) molecule on the surface of antigen presenting cells (APCs) 3. Moreover, MAIT cells can be activated by various inflammatory cytokines, such as IL-12, IL-18, in a MR1-independent manner. Therefore, MAIT cells can be considered both non-specific immune and acquired immune cells.

## 2. Characteristics of MAIT cells

MAIT cells express a semi-invariant TCR-α chain (made of an invariant Vα7.2-Jα33 in humans and Vα19-Jα33 in mice) and a limited TCRβ chain 4, 5. In 1993, Porcelli et al. found that αβCD4^-^CD8^-^T cells existed in peripheral blood of healthy volunteers and selectively expressed invariant TCR α chain 6. One of them called iNKT cells, which was composed of Vα24 and Jα18 gene fragments (Vα14 and Jα18 in mice). The other one was composed of Vα7.2 and Jα33 gene fragments (V α 19 and J α 33 in mice). In 1999, Tilloy et al. 7 validated that the constant expression of TCRα Vα7.2/Vα19-Jα33 resulted from a subset of certain T cells. Until 2003, Treiner et al. 4 found this new T cell population originated from the intestinal mucosa, defined as the mucosal-associated invariant T cells. Once recognizing the nonclassical MHC class IB molecule (MR1) presented by antigen presenting cells (APCs), they could produce a variety of cytokines, involving directly or indirectly in immune responses.

MAIT cells are important lymphocyte subsets, representing 0.1-10% of total T cells 2, 8, 9. The most common subset population of MAIT cells is CD8^+^ effector memory phenotype. Double-negative MAIT cells (CD4^-^CD8^-^) also hold a certain proportion. However, CD4^+^ MAIT cells are relatively rare 10. Notably, most CD8^+^ MAIT cells express the homodimer CD8αα and only a few express CD8αβ 11. MAIT cells are absent in germ-free mice. The latest research explained possible mechanisms. It elucidated that vitamin B2 precursor derivatives 5-OP-RU produced by commensal bacteria entered thymus through mucosal barrier, and induced the maturation of MAIT cells through TCR signal 12. Considering that exogenous 5-OP-RU could be captured and presented by thymic cells. It is of great significance for clinical and drug research. Nevertheless, MAIT cells are rare in laboratory strains of mice (C57BL/6 and BALB/c). The proportion is approximate to 0.6% of T cells in mice liver 13. Recently, the soluble tetramerized MR1 molecules, refolded with 5-OP-RU Ag can be used to detect MAIT cells in both blood and tissues 13-15. Meanwhile, many experimental studies used the MAIT cell-deficient mice (MR1^-/-^) 4 and the mice with high frequency of MAIT cell (Vα19TCRTg) 16, 17 to explore the possible mechanisms of different diseases. However, two models cannot completely represent human MAIT cells. Firstly, MAIT cells are similar in WT mice and humans. However, the distribution of MAIT cells is lower compared in laboratory mouse strains with human. Secondly, MAIT cells exhibit different phenotype in the TCR transgenic mice, which cannot reflect the normal biology 13. The result of using transgenic mice to study MAIT cells still need to be further verified in human samples. Besides, the MR1^-/-^ mice were not completely absent 18, 19. Moreover, MR1 restricted T cells are more diverse than MAIT cells 20, so MR1-deficient mice are not exactly the same as MAIT-deficient mice model. Recent research also discovered CAST/EiJ mouse strain which had more MAIT cells 21 and Traj33KO mice in which MAIT cells were markedly diminished 22. However, their application in different disease models still requires further experimental researches.

**Figure 1:**
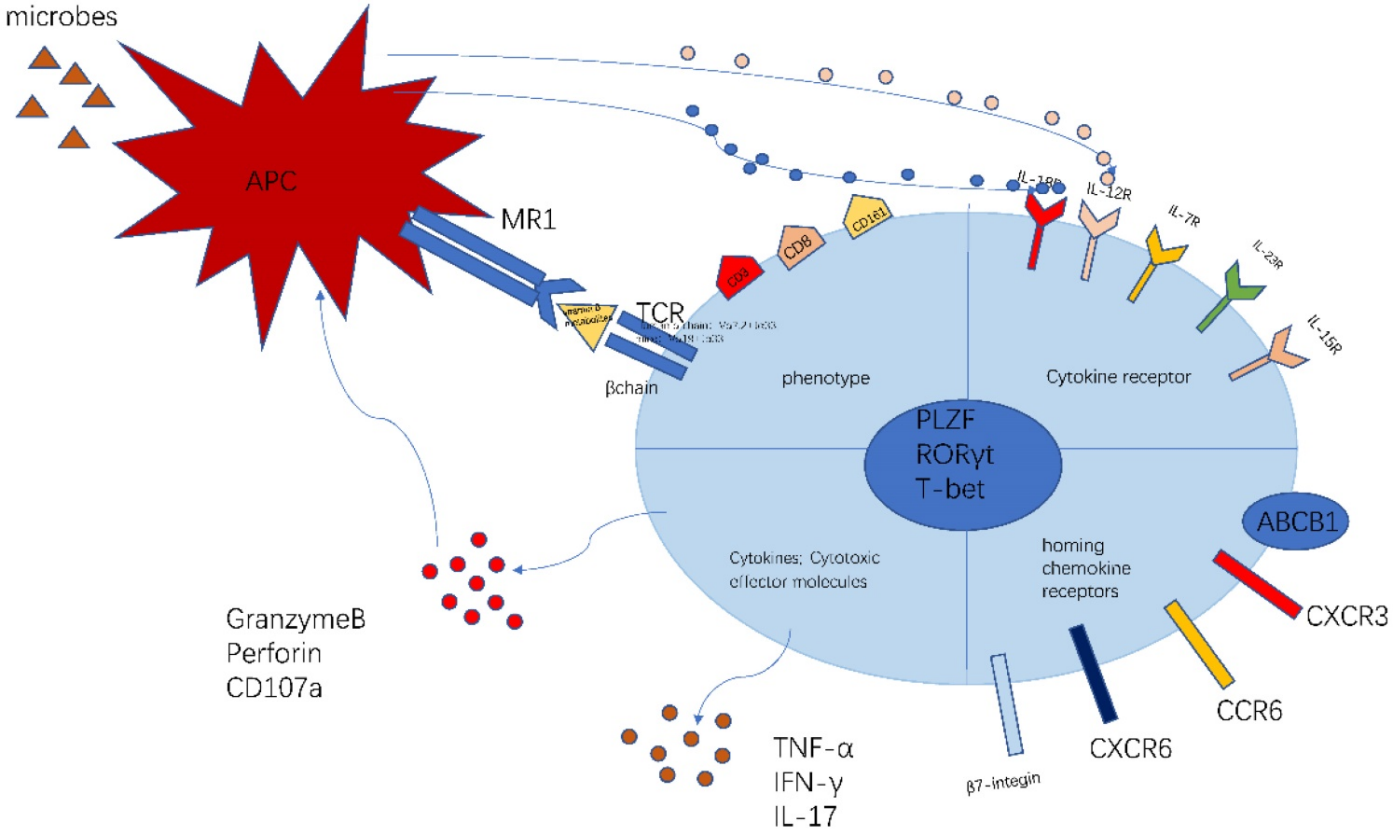
** The characteristics of MAIT cell.** MAIT cells are defined as CD3^+^CD161^+^Vα7.2^+^ lymphocytes. Most MAIT cells are CD8^+^T subset. The MR1-5-OP-RU and TCRβ can define MAIT cells. MAIT cells possess homing receptors CCR6, CXCR6, β7-integrins and CXCR3 to migrate to the liver, intestines and some inflammatory tissues. MAIT cells express cytokine receptors IL-12R and IL-18R, which can be activated by different cytokines. Meanwhile, MAIT cells have the multidrug resistance transporter (ABCB1). Once being activated, MAIT cells produce Th1 and Th17 cytokines, such as TNF-α,IFN-γ,IL-17. Meanwhile MAIT cells release granzyme B and perforin to kill the infected cells. This picture was adapted from the reference 91.

The phenotype of MAIT cell is essential and important to identify their function (Figure 1). The previous researches have proved that MAIT cells express PLZF, RORγt and T-bet transcription factor 11, 23, consequently regulating the rapid effector function. Besides, MAIT cells also possess a series of specific homing receptors, such as CCR6, CXCR6 2, beta7-integrins, CXCR3 24, which targets MAIT cells to the liver, intestines and some inflammatory tissues. Moreover, MAIT cells express a series of innate cytokine receptors such as IL-12R, IL-18R and IL-23R, which is of great importance to respond to these cytokines in pathological conditions without TCR ligation. Notably, different modes of activation express distinct function phenotype. TCR-activated MAIT cells resembled Tc17 phenotype while cytokines-activated MAIT cells were similar to the Tc1 phenotype 25. Most MAIT cells express the multidrug resistance transporter (ABCB1), which efflux xenobiotics and are resistant to chemotherapy 8, 26. Upon activation, MAIT cells secrete Th1 and Th17 cytokines, which is involved in infectious and non-infectious inflammatory diseases 27. In addition, the recent study discovered the chronic stimulation promoted MAIT cells to produce Th2 cytokines 28. Meanwhile, MAIT cells regulate cytolytic capacity by secreting granzyme B and perforin 23, 29. However, the recent research also proposed a new opinion that bacterial superantigens turn MAIT cells against each other. Animal models and human cells were used to confirm that MAIT cells became hyperreactive and exhausted when exposed to bacterial superantigens. This exhaustion led to immunosuppression, which increased the risk of secondary opportunistic infections and caused fatal consequences 30.

As an innate immune cell, MAIT cells are playing a pivotal role in immune network. They promoted inflammatory monocyte differentiate into dendritic cells^31^, as well as matured monocyte-derived and primary DCs, which helped coordinating the adaptive immune response^32^. During the phase of infection, MAIT cells were necessary for cytokine production and recruiting activated CD4^+^ and CD8^ +^T cells 33. Moreover, MAIT cells may help improve B-cell responses and promote the reactivation and differentiation of memory B cells 34.

The activation and function of MAIT cells could be influenced by multiple factors. As we all know, bacteria species activate MAIT cells in a MR1-dependent manner 35. This process required both intact bacterial to access an acidified endosomal compartment and the help with APC through NF-kB or interferon signaling pathways 36. The study has verified bacteria resulted in the reduction of MAIT cells 24. Furthermore, bacteria become opsonized with IgG and complement, which triggered TNF production of macrophages, thus enhancing the MAIT cell responsiveness 37. A recent study reported endosomal TLR9 participated in antigen presentation process 38. Meanwhile, MR1 in different cell lines used an endocytic pathway to activate MAIT cells, independent of bacteria 39. Cytokines also activated and stimulated MAIT proliferation 40, 41. Particularly, some studies demonstrated that MAIT cells respond to TLR8-mediated activation by inducing a strong immune response 42. In addition to the TLR8 agonist, some drugs, such as salicylate, regulated the activity of MAIT cells 43. Once activated, MAIT cells show their effector functions, including producing cytokines and inducing cytolysis. Sometimes these processes were accompanied with the increasing uptake of glucose 44. Furthermore, it was also reported that the mTORC1 signaling was relevant to the glycolysis of MAIT cells, leading to altered MAIT cell responses 45. The recent study discovered TCR and cytokines can efficiently and synergistically activate human MAIT cells, including TL1A. Besides, the inhibition of TL1A can limit inflammation. Interestingly, TCR-dependent triggering of MAIT cells can play an important role in tissue repair 46. At the same time, another study also confirmed activated MAIT cells expressed a tissue repair phenotype in both human and mice 47.

It was interesting to note that most studies discovered the obvious decrease in circulating MAIT cells. However, the reason for this change is less clear. Some studies found the peripheral MAIT cells were age-related and changed with age 48-50. The most common explanation for the reduction was attributed to the apoptosis 17, 51. Controversially, some researchers found that the decrease may be due to the cell exhaustion 52-54. In addition, the specific microenvironment may ascribe to this change, possibly through activation-induced cell death, such as microbial translocation, chronic inflammatory conditions 55. As well, with the tissue specimen, some researchers found that the circulating MAIT cells may migrate to the inflamed tissues, with the upregulation of chemokine receptors 9, 52. Besides, the repetitive cytokine stimulation also led to the decrease in MAIT cell frequencies 56, 57.

Recently, increasing studies focus on MAIT cells. Some research has shown that MAIT cells played a protective role in various infections, such as bacteria 27, 58, viruses 59. It was also demonstrated that MAIT cells were involved in tumors 60, 61. Meanwhile, flawed MAIT cells contributed to the progression of the pathogenesis in immune diseases 62, 63. Interestingly, MAIT cells also played a necessary role in metabolic diseases 45, 64 and parasitic diseases 65.

Due to the large amount of MAIT cells in the human liver, mounting evidence suggested that MAIT cells played complex roles in regulating different liver disease. Jeffery et al. found the phenotype and role of MAIT cells in various etiological chronic liver diseases, and analyzed the localization of MAIT cells in liver, which proved MAIT cells mainly concentrated in the bile ducts, portal tracts and hepatic sinusoids 9. Hence, other researches also examined the distribution of MAIT cells with liver samples, demonstrating MAIT cells preferentially resided in portal tracts other than in the parenchyma 66. Besides, MAIT cells were associated with liver fibrosis. Some studies found MAIT cells could accumulate in fibrotic septate in diseased livers 57. MAIT cells in the liver may be induced apoptosis. Meanwhile they may redistribute in the liver. Therefore, it is not yet uncertain what changes MAIT cells may happen in liver.

## 3. MAIT cells in liver diseases

### Alcoholic liver disease

Currently, Alcoholic liver disease (ALD) has gradually become one of main causes of chronic liver disease 67. A latest research demonstrated that the level of consumption which can reduce the health loss is zero by analysis for the Global Burden of Disease Study 68. The basic stages of alcoholic liver disease include liver inflammation, hepatocyte regeneration disorder and translocation bacteria 68. We concluded that one of the major functions of MAIT cells is to inhibit bacteria. Riva et al. 24 explored the relationship between liver, intestinal immunity and the effect of MAIT cells in ALD. They emphasized that it was more evident in severe alcoholic hepatitis (SAH) patients with severe bacterial infection, accompanying with the obvious reduction in MAIT cells. In addition, they also confirmed that it was fecal bacteria that induced functional impairments and reduction of MAIT cells from ALD. However, they just put attention to the SAH and ARC patients; we can't imagine what changes would happen in the patients involving binge drinking, alcoholic fatty liver and chronic drinking without liver disease. Besides, the depletion of circulating MAIT cells contributed to the caspase-dependent apoptosis in this article. What about other programmed or accidental cell death elucidating this phenomenon?

Meanwhile, we could summarize that short-term drinking abstinence had no effect in MAIT cell levels. Nevertheless, Wei Li et al. 70 explored that long-term alcohol abstinence partially reversed MAIT cell abnormalities. However, the residual MAIT cells in alcoholic hepatitis (AH) patients were highly activated with high levels of activation markers CD69, CD38, HLA-DR. Unlike the previous study, Wei Li reported that MAIT cells can express higher levels of exhaustion marker PD-1, not observing an increase in apoptotic of MAIT cells in AH patients.

In addition to their antibacterial functions, MAIT cells are a profibrogenic immune cell population 17. This research showed that both blood MAIT cells and liver MAIT cells reduced in the alcoholic cirrhosis. This is different from the previous study which found that intrahepatic MAIT cells in ALD were not deleted. Pushpa Hegde demonstrated that intrahepatic MAIT cells accumulated in the fibrotic septa 17. In vivo and vitro experiments validated the profibrogenic properties of MAIT cells. Furthermore, their findings suggested that activated MAIT cells promoted the transformation of hepatic myofibroblasts to a proinflammatory phenotype, characterized by increasing production of IL-8 and IL-6.

These studies opened a new direction on the fields of MAIT cells. Bin Gao et al. 71 confirmed MAIT cells likely exerted an important role in controlling the bacterial infection though MAIT cells had defects in the frequency and function among ASH patients. He also suggested that there are more aspects to explore. We can study the intrahepatic and gut MAIT cells in SAH patients in detail. Then, we can relate the gut-liver axis with MAIT cells. Finally, we can also study bacteria how to affect MAIT cells in ALD patients. In addition to these thoughts, we need to make some researches to investigate the characteristics of MAIT cells in patient at different stages of ALD disease. We should learn more about the relationship between the MAIT cells and the immune network in the liver.

### Non-alcoholic fatty liver disease

It is reported that 25% adult has non-alcoholic fatty liver disease (NAFLD) 72. NAFLD is also a chronic inflammation associated with a high-fat diet and an imbalance in intestinal microbial homeostasis. As well, NAFLD is generally closely related to metabolic diseases, most patients have insulin resistance as well as lipid metabolism disorder. Lately, YM. Li et al. 73 indicated that MAIT cells may have a positive effect on NAFLD patients through producing regulatory cytokines including IL-4 and IL-10, resulting in anti-inflammatory macrophage polarization. MAIT cells regulate immune response and reduce the liver inflammation. In their study, they found high free fatty acids could increase MR1 expression in Kupffer cells. The number of MAIT cells in the liver positively correlated with NAFLD activity score. As well, there was a connection between the circulating MAIT cells frequency and clinical parameters relevant to the metabolic diseases. Comparing with the MAIT cell deficient MCD mice, we confirmed MAIT cells can alleviate inflammation. However, what accompanied is that MAIT cells may contribute to the liver fibrosis 17. It is necessary to assess the advantages and disadvantages of MAIT cells in NAFLD patients. Meanwhile, we found MAIT cells attenuated lipid deposition in the liver. Interestingly, MAIT cells could accumulate in the adipose tissue among obese patients 64. The relationship between the distribution and function of MAIT cells with lipid metabolism may become new research directions.

Considering that NAFLD encompasses a series of disorders, such as diabetes, obese, hyperlipidemia, we also put our insight into the roles of MAIT cells in the metabolic disease. Rouxel et al. 52 explained the cytotoxic and regulatory roles of MAIT cells in type 1 diabetes. They highlighted the MAIT cells are of importance in the maintenance of gut integrity and the effect on anti-islet autoimmune response. By analyzing MAIT cells in patients, they deemed circulating MAIT cells as a new biomarker of progression in T1D. Besides, the animal research confirmed the effect of MAIT cells was distinct in the pancreas and the gut mucosa. In pancreas, MAIT cells correlated with the diseases progress. They could produce granzyme B and IFN-γ, which directly killed β-cells and aggravated the diseases. In contrary, MAIT cells showed their friendly side in the intestine. MAIT cells could secret IL-17A and IL-22 to increase the tight-junction proteins. Therefore, it still needs to investigate the regulatory mechanism of MAIT cells in diabetes. Nowadays, increasing attention has been paid to the study of dysbiosis of the microbiome and bacterial overgrowth in patients with NAFLD. Importantly, MAIT cells participate in the protection of intestinal barrier. Besides, they are related to the gut dysbiosis. So, it will be promising to make a further research on the link between MAIT cells with the gut microbiota in NAFLD patients.

### Autoimmune liver disease

Autoimmune immune liver disease (AILD) is characterized by chronic inflammation in the liver which is caused by autoimmune response. AILD contains autoimmune hepatitis (AIH), primary biliary cholangitis (PBC), primary sclerosing cholangitis and any overlap syndrome between the three diseases. AIH targets hepatocytes and is mainly related to hepatocyte injury. The objective of autoimmune attack of PBC and PSC are bile duct epithelial cell, which are characterized by cholestasis. However, those diseases are all accompanied by inflammation and fibrosis 74.

Böttcher et al. 57 found that MAIT cells were depleted in both peripheral blood and liver tissue in AILD patients. Meanwhile, intrahepatic MAIT cells tended to decrease with increasing fibrosis stage. The decrease was associated with long-term exposure to cytokines and bacterial antigens, which down-regulated the T-bet and EOMEs, resulting in cell exhaustion. Meanwhile, activated MAIT cells could secrete IL-17A, which induced HSC proliferation. Renand et al. 75 put their attention on Type 1 Autoimmune Hepatitis. They found the circulating MAIT cells may recruit to the inflammatory liver, dominantly accumulating in the portal tract. They also summarized the granzyme B expression by MAIT cells correlated with the fibrosis. Setsu et al. 76 focused on the PBC patients. MAIT cells were obviously reduced, along with expressing lower levels of activation markers CD69 and IL-7 receptor. The cytokine productions of MAIT cells also were impaired. After ursodeoxycholic acid (UDCA) treatment, the aforementioned changes were not fully recovered, which may account for the persistent inflammation in the liver. Jiang et al. 66 discovered the increase in MAIT cells in liver tissues among PBC patients. They also verified that abnormalities of circulating MAIT cells from PBC patients can be attenuated after 6 months of UDCA treatment. Importantly, they connected the effect of MAIT cells with cholic acid, which is essential in the pathological mechanism of PBC. The cholic acid could stimulate IL-7 expression in hepatocytes through activation of FXR. The increasing IL-7 induced phosphorylation of STAT5 in MAIT cells, which enhanced cytokine production. Seth et al. 77 assessed the effect of MAIT cells on PSC, which is known for the injury of bile ducts. They explored the MAIT cells in human bile ducts through biliary brush samples, which obtained from endoscopic retrograde cholangiopancreatography (ERCP). They confirmed an obvious increase in MAIT cells. However, they did not further evaluate the function of MAIT cells in bile duct. In agreement with previous reports, they found MAIT cells were lost from circulation and presented an activated and impaired phenotype.

According to these scientific researches, we can conclude MAIT cells were depleted in the circulating blood and showed an activated and impaired phenotype. However, the change in intrahepatic MAIT cells was controversial. Also, in the context of immune liver diseases, are there any other effects except antimicrobial and profibrogenic effects? Maybe we should focus more on the mechanism of immune responses and cholestasis with MAIT cells. It is important to explore the mechanism they communicate with each other.

### Viral hepatitis

Chronic hepatitis B virus infection is a major global health threat 78. The IFN-γ is an important antiviral cytokine. Intrahepatic MAIT cells are the most important innate effector cells secreting IFN-γ^42^. Many researches concentrated on the production of IFN-γ and granzyme B of MAIT cells in chronic viral liver diseases. Yong et al. 79 investigated HBV DNA^+^ and HBV DNA^-^ patients, they discovered peripheral MAIT cells significantly reduced and produced less granzyme B and IFN-γ, which correlated with the levels of CD69 expression. However, in another study by Boeijen 80, MAIT cells were not depleted in blood of chronic hepatitis B patients. This discrepancy may be attributed to many factors. The possible causes may include the number of patients in the experimental group, the different phase of disease and the limitation of individual difference. We still need a long-term and comprehensive study. However, Yong et al. 53 also indicated that the decrease may be driven by HBV-induced chronic immune activation. The high expression of PD-1 on MAIT cells could predict plasma HBV-DNA levels. The study also gave us another direction to further investigate, on which we can block co-inhibitory molecule expression in order to restore function of MAIT cells.

Hepatitis C infection is also a research hotspot. Bolte et al. 81 analyzed chronic HCV infection patients during antiviral therapy. The frequency of MAIT cells in both blood and liver were decreased before the therapy. The decrease of MAIT cells were related to chronic inflammation in the liver, which was caused by chronic HCV infection. After 4 weeks later of antiviral therapy, the frequency of hepatic MAIT cells increased with the decrease of hepatic inflammation. However, the change of circulating MAIT cells was undiscovered. Additionally, MAIT cells in the liver were associated with the decrease in their activation status and cytotoxic effector function by 4 weeks antiviral therapy. Besides, MAIT cells also expressed a high level of degranulation marker CD107a. However, the ability to respond to E. coli stimulation in chronic HCV infection decreased, which demonstrated that their defense mechanisms against bacterial infection was impaired. Likewise, Hengst et al. 82 reported the phenotype and function of MAIT cells in chronic HCV patients who were observed within IFN-free therapy. However, they just collected the blood sample. They confirmed MAIT cells were severely reduced and nonreversible with IFN-free therapy. Also, the higher expression of granzyme B, HLA-DR, PD-1 and CD69 represented the activated and exhausted phenotype. Importantly, the impaired response to MR1-dependent was once again confirmed. Lately, there were also some articles about how HCV/HIV co-infection impacted MAIT cells 83, 84, these studies only seized on the changes of MAIT cells, including frequency and cytokine production. Interestingly, the latest research on the relationship between MAIT cells and HIV- and/or HCV-infected patients found that the mono- and co- viral infection caused dysbiosis, which hampered the antibacterial function of MAIT cells. They also suggested the reduction of MAIT cells in the blood may ascribe to the down-regulation of CD161 85. Meanwhile, some experts focused their attention on HDV infection 56. In their experiment, in both peripheral blood and liver, MAIT cells dramatically declined.

In conclusion, MAIT cells showed impairment of immune response function. In parallel to the reduction of MAIT cells, the increasing IL-12 and IL-18 may contribute to the MAIT cell activation, loss and cell death. Therefore, we should demand further investigation to make sure what real roles do the MAIT cells play. Whether MAIT cells can be a new therapeutic target by enhancing the release of IFN-γ.

### Liver cancer

Liver cancer, especially hepatocellular carcinoma, is the fourth cause of cancer-related deaths worldwide 86. All kinds of chronic diseases may eventually develop into liver cancer. The occurrence and development of liver cancer involves a variety of complex mechanisms. Among different mechanisms, the role of cannabinoid Receptor1 (CB1R) cannot be ignored 87. The upregulation of cancer-promoting FOXM1 gene by CB1R was via a G_i/o_/CAMP/CREBP pathway. Then FOXM1 can induce IDO2 expression and activation, which could upregulate Treg cells and proangiogenic genes. However, the management of liver cancer is difficult and complex. The treatment includes surgical resection, transplantation, percutaneous local ablation, transarterial embolization, radiotherapy and systemic pharmacological treatment 88. Just as the research in CB1R, the tumor immune microenvironment and the inflammatory state in liver contribute to the progression of the diseases. Recently, increasing studies focused on the tumor immunotherapy, which control and remove tumors. Furthermore, immunotherapy can restart and maintain the tumor-immune cycle and restore the normal anti-tumor immune response. MengDuan et al. 89 focused on the relationship between hepatocellular carcinoma and MAIT cells, trying to reveal new approaches for cancer. They found that high density of tumor-infiltrating MAIT cells may be associated with unfavorable outcomes in HCC patients. In their study, they demonstrated MAIT cells were depleted in the tumor samples and expressed higher levels of immune checkpoints, such as PD-1, which proved the reduction of MAIT cells contributed to the immune exhaustion rather than apoptosis. In the context of tumor microenvironment, tumor-derived MAIT cells secreted less IFN-γ, IL-17 and granzyme B, perforin, but they secreted more IL-8, which is of great importance in promoting tumor angiogenesis and progression. Besides, they co-cultured MAIT cells from healthy people PBMC, peritumor liver tissues and tumor-infiltrating with HCC cells. They concluded that normal MAIT cells could induce apoptosis of HCC cells, but their ability impaired in tumor environment. So, the future researches need to concentrate on how to modify MAIT cells to exert the anti-tumor efficiency. In addition to the primary liver cancer, the hepatic metastases are also an intractable problem in clinics. Except the surgical resection, the immunotherapy opens up a new direction of treatment. Shaler et al. 90 observed the frequency of MAIT cells were less abundant in hepatic metastases of colorectal carcinoma, while the function of MAIT cells were impaired as well. Besides, dysfunction of MAIT cell is not related to chemotherapy, for the high expression of MDR-1^8^, ^26^.

According to above results, we propose MAIT cells provide a new therapeutic target for the treatment of cancer. The mechanisms of MAIT cells involved in cancer progression need to be further studied. More basic studies are needed to figure out MAIT cells agonist. It is of great importance to combine MAIT cells with chemotherapeutic agents.

## 4. Conclusion

From the previous studies, infinite possibilities of MAIT cells have been discussed. However, due to the lack of liver samples, it is still unclear about the changes and function of intrahepatic MAIT cells. We cannot make the unified conclusion on how MAIT cells change in chronic liver injury and what mechanisms they participate in. Still, there are many cell types in the liver including hepatocytes, liver sinusoidal endothelial cells, NK cells and so on. There is a complex immune network. It is also a challenge for us to consider MAIT cells how to communicate with different cell types and change with the changing cells in the context of liver diseases. Considering the distribution of MAIT cells, they can exert its different effects on different organs. It will be interesting to get some insight into crosstalk between different organs, such as gut-liver axis, fat-liver axis. In different kinds of acute and chronic liver diseases, liver inflammation, immune disorders and microbiome dysbiosis can aggravate the progression of the disease. MAIT cell as an important member of immune cells, they can play important roles in the above-mentioned situation. We also need to distinguish whether MAIT cells in liver are our friends or foes. Therefore, it is of great importance to understand how to balance the advantages and disadvantages of MAIT cells. Some researches focus on the fundamental research of MAIT cells activation. We can learn that TLR8 agonists and salicylate can activate MAIT cells. Besides, with the discovery of cell exhaustion, if the immune checkpoint inhibitors can be the new therapeutic direction. Accompanying with the more studies about the agonists, we may modulate the MAIT cells in order to adapt to the different micro-environment. Along this line of consideration, whether MAIT cells can be used as biological markers for early diagnosis and prevention needs further study. Moreover, the mechanism why MAIT cells decrease in peripheral blood is still unclear, and we also need to explore new methods that can change the frequency and improve the function of MAIT cells. In view of limitation of studies where mice are totally different than people in functionality of MAIT, the suitable mouse model still need to be investigated and more human tissue samples also need to be studied.

In summary, MAIT cells can be a new and effective treatment for the liver disease. We speculate that in the next five years, the more clinical hepatic MAIT cell researches to characterize human MAIT cells. More researches to find out the detailed causes of MAIT cell changes. Finding the way to activate or inhibit MAIT cells in order to adapt to the different disease state. MAIT cells in combination with drugs treatment will solve the difficulties in the complication of chronic liver diseases.

## Figures and Tables

**Figure 2:**
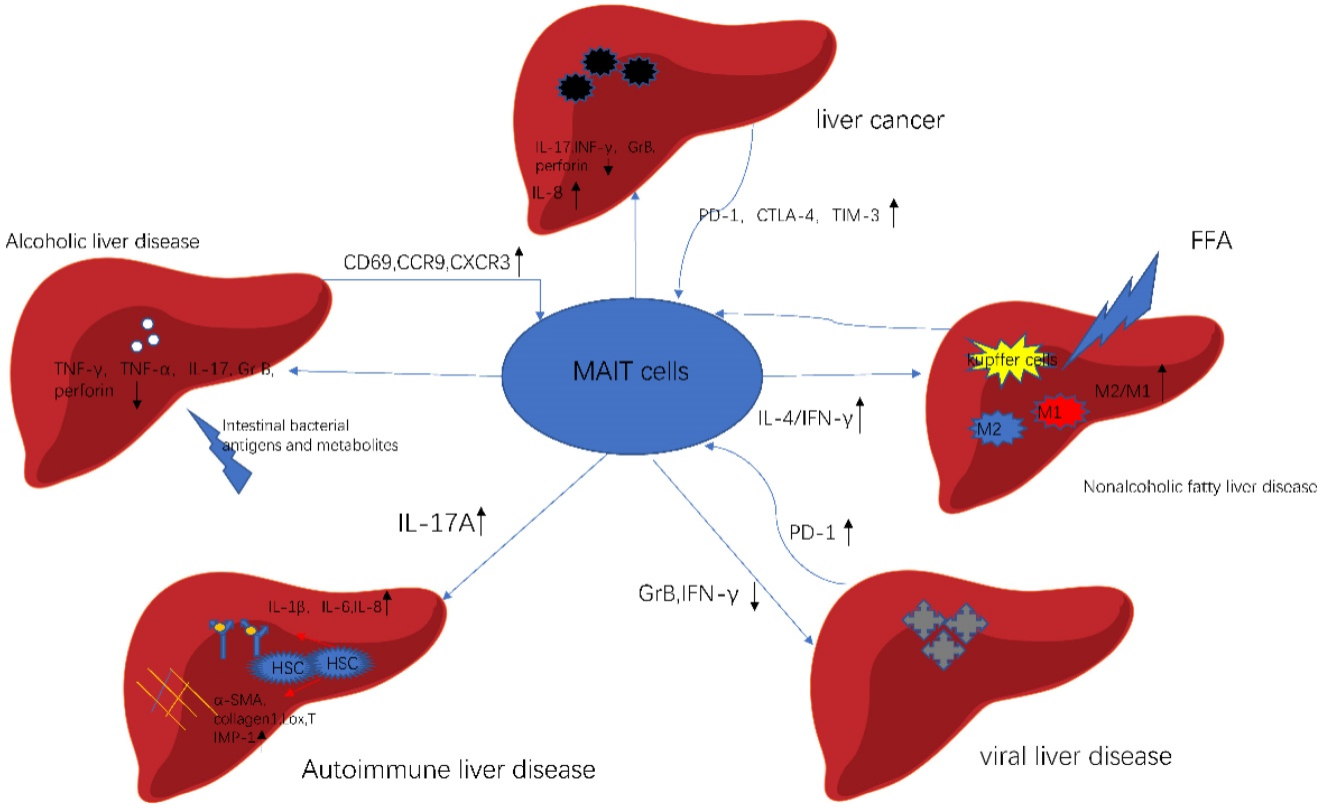
** MAIT cells in different liver diseases.** In alcoholic liver disease, gut bacterial directly affect the function of MAIT cells. In nonalcoholic fatty liver disease, FFA stimulates Kupffer cells, which activates MAIT cells to produce IL-4. As a result, activated MAIT cells promote macrophage to polarize. In autoimmune liver disease, MAIT cells are a profibrogenic and proinflammatory cell population. In viral liver disease, MAIT cells exhibit cell exhaustion and impaired effector function. In liver cancer, MAIT cells show activated and exhausted phenotype. Meanwhile, MAIT cells enhance the progression of cancer. This picture was adapted from the reference 92.

**Table 1:** MAIT cells in liver diseases

Disease	frequency		phenotype		function		Reference
	blood	tissue	blood	tissue	blood	tissue	
Alcoholic liver diseases							
AH/SAH	↓	-	Beta7-integrin- CXCR3/CCR9/CX3CR1/IL7R- IL-18R/caspase3/CD26/DPP4^high^ - CD69/HLA-DR↑ LAG3/Ki67/CD57/ TIM-3- PD-1↑/-	α4/αE integrins↑ CXCL10↑	IFN-γ/TNF-α/ Perforin- IL-17↓/CD107a↓ RORC/RORrt/ZBTB16/PLZF/Eomes/T-bet↓ GranzymeB-/↑	RORC/RORrt/ZBTB16/PLZF/Eomes↓	24
HDC	↓		Caspase3- CD69/CD38/TIM-3/PD-1-				70
ARC	↓	↓	CD25/CD69↑Ki-67↑ TIM-3/PD-1/Bcl-2-	TIM-3/PD-1/HLA-DR↑ CD25/CD69↓	IL-17↑GranzymeB↓ IFN-γ/TNF-	IL-17↑ GranzymeB/IFN-γ/TNF-	17
Non-alcoholic liver disease	↓		CD69/PD-1/CXCR6↑ CCR5^high^-		IFN-γ/TNF-α↓IL-4↑ IL-10-		73
Autoimmune liver disease							
AIH	↓	↑/-	CD69↓		GranzymeB↑IFN-γ↓	Granzyme B↑	57,75
PSC	↓	↓/ ↑/-	CD69/CD56/NKG2D/HLA-DR/CTLA-4/CD39/CD38↑ CD28/CD127/CXCR6↓ PD-1↑/-		CD107a/TNF/IFN-γ↓		57,77
PBC	↓	↑/ ↓/-	CD38/CD25/ Caspase3/VLA-4/ TIM-3/CXCR6↑ CD69/ IL-7R/IL-18R↓ CCR6↑/- CCR5/ NKG2D-	CD69↑ IL-7R/IL-18R/NKG2D-	PMA stimulation: IL-17/GranzymeB↑ IFN-γ/TNF↓ TCR stimulation: IL-17/ TNF-α/IFN-γ↑ GranzymeB-		57,66,76
Viral hepatitis							
HBV	↓/-	CD69↓/- CD38/PD-1↑ CD25/2B4- CTLA-4/ TIM-3/ HLA-DR↑/-		Granzyme B↑/↓/- IFN-γ/ TNF-α-/↓ Perforin-			53,79,80
HCV/HIV	↓	↓/-	CD69/CD38/HLA-DR/PD-1/CD107a↑		IFN-γ↓TNF-α↓GranzymeB↓/↑ IL-17-		81-85
HDV	↓	↓	CD38/HLA-DR/PD-1↑ CD28/CD127↓ Ki-67/PLZF/Eomes/T-bet-		IFN-γ↑ CD107a/GranzymeB↓		56
Liver cancer							
Hepatocellular carcinoma	↓	↓	CCR7^-^CD45^-^RA^-^CD45RO^+^CD95^+^ CD38/HLA-DR- CD160↓ CTLA4/TIM-3/PD-1↑	CD28/CD127↓ CD38/HLA-DR↑ CCR6/CXCR6/CCR9↓CXCR3/CCR2↑CCR5^high^- CTLA4/TIM-3/PD-1↑ BAX/BID/Bcl-2-	IFN-γ/IL-17↓	IFN-γ/IL-17/ GrB/perforin↓ IL-8↑ IL-4/IL-10/IL-22-	89
Hepatic metastasis	↓	↓		IL-12R/IL-18R-		IFN-γ↓	90

SAH: severe alcoholic hepatitis; ARC: Alcohol-related liver cirrhosis; AH: alcoholic hepatitis; HDC: heavy drinkers without overt liver disease; AIH: autoimmune hepatitis; PSC: primary sclerosing cholangitis; PBC: primary biliary cholangitis; EOMES: Eomesodermin; PLZF: promyelocytic leukemia zinc finger; RORγ T: retinoic acid-related orphan receptor γT; T-bet: T-box expresses; TCR: T cell receptor; PMA: phorbol 12-myristate 13-acetate; IFN: interferon; IL: interleukin; TNF: tumor necrosis factor; CCR: CC-chemokine receptor;PD-1: programmed cell death 1;TIM3: T cell immunoglobulin and mucin domain-containg; ↑increase↓decrease**-** comparable; compared with blood or tissue from healthy individuals or with unaffected tissues.
